# Effects of dietary coenzyme Q10 supplementation during gestation on the embryonic survival and reproductive performance of high-parity sows

**DOI:** 10.1186/s40104-023-00879-4

**Published:** 2023-06-02

**Authors:** Shanchuan Cao, Honglin Yan, Wenjie Tang, Hongfu Zhang, Jingbo Liu

**Affiliations:** 1 School of Life Science and Engineering, Southwest University of Science and Technology, Mianyang, 621010 China; 2 Department of Animal Resource and Science, Dankook University, Cheonan, 31116 Korea; 3 Livestock and Poultry Biological Products Key Laboratory of Sichuan Province, Sichuan Animtech Feed Co., Ltd., Chengdu, 610066 China; 4 State Key Laboratory of Animal Nutrition, Institute of Animal Sciences, Chinese Academy of Agricultural Sciences, Beijing, 100193 China

**Keywords:** Coenzyme Q10, Embryonic survival, Oxidative stress, Parity, Sows

## Abstract

**Background:**

Fertility declines in high-parity sows. This study investigated whether parity-dependent declines in embryonic survival and reproductive performance could be restored by dietary coenzyme Q10 (CoQ10) supplementation.

**Methods:**

Two experiments were performed. In Exp. 1, 30 young sows that had completed their 2^nd^ parity and 30 high-parity sows that had completed their 10^th^ parity, were fed either a control diet (CON) or a CON diet supplemented with 1 g/kg CoQ10 (+ CoQ10) from mating until slaughter at day 28 of gestation. In Exp. 2, a total of 314 post-weaning sows with two to nine parities were fed the CON or + CoQ10 diets from mating throughout gestation.

**Results:**

In Exp. 1, both young and high-parity sows had a similar number of corpora lutea, but high-parity sows had lower plasma CoQ10 concentrations, down-regulated genes involved with de novo CoQ10 synthesis in the endometrium tissues, and greater levels of oxidative stress markers in plasma and endometrium tissues. High-parity sows had fewer total embryos and alive embryos, lower embryonic survival, and greater embryo mortality than young sows. Dietary CoQ10 supplementation increased the number of live embryos and the embryonic survival rate to levels similar to those of young sows, as well as lowering the levels of oxidative stress markers. In Exp. 2, sows showed a parity-dependent decline in plasma CoQ10 levels, and sows with more than four parities showed a progressive decline in the number of total births, live births, and piglets born effective. Dietary supplementation with CoQ10 increased the number of total births, live births, and born effective, and decreased the intra-litter covariation coefficients and the percentage of sows requiring farrowing assistance during parturition.

**Conclusions:**

Dietary CoQ10 supplementation can improve the embryonic survival and reproductive performance of gestating sows with high parity, probably by improving the development of uterine function.

## Introduction

Two factors determine the primary economic value of a sow: the number of weaned piglets provided per sow per year and a sow’s lifetime of service, which together determine a sow’s lifetime productive performance [1]. Chinese culling data show that sows are culled due to diseases relating to reproduction, common diseases, lameness, failure to conceive, anestrus beyond seven days postweaning, and low litter size, all of which significantly increase the unplanned culling of sows [2]. Increasing attention has been given to lifetime reproductive performance in the pig rearing industry and the development of management solutions to extend the reproductive span of sows.

Reproduction is an energy-expensive process which requires a consistent supply of adenosine triphosphate (ATP) to maintain normal cellular function in the reproductive tissues such as the ovaries and uterus [3]. However, there is an age-dependent decline in cellular energetics which drives early reproductive failure.

Coenzyme Q10 (CoQ10) is a fat-soluble quinone ring compound with a structure similar to vitamin K and is a strong free radical scavenger acting as an antioxidant component of the mitochondrial respiratory chain in various body tissues [4]. Mammals and eukaryotes can synthesize CoQ10 de novo via two key rate-limiting enzymes encoded by the *PDSS-2* (decaprenyl diphosphate synthase subunit 2) and *COQ-6* genes [5, 6]. However, the expression of *PDSS-2* and *COQ-6* has been observed to decline in the oocytes in the reproductive tissues of females in both mice and humans, leading to impaired mitochondrial function in the ovarian follicles and oocytes [6, 7]. Under such conditions, exogenous administration of CoQ10 to mice rescued this diminished fertility in older individuals [6–8]. In addition, supplementation with CoQ10 during in vitro maturation of porcine oocytes improved mitochondrial function and protected against the negative effects of oxidative stress on developmental competence [9, 10]. CoQ10 is a crystalline powder insoluble in water and its absorption process and bioavailability was affected by several factors such as carrier lipids and solubilization [11]. However, it remains unclear whether dietary supplementation with CoQ10 could exert a positive effect on the reproductive performance of sows in vivo. This study set out to investigate the age-dependent changes in CoQ10 production in sows, and to test the hypothesis that dietary supplementation with CoQ10 in sows of various parities could provide beneficial effects on reproductive performance and lower the culling ratio of sows.

## Materials and methods

### General

The Animal Care and Use Committee of the Southwest University of Science and Technology (Mianyang, China) approved the experimental procedures used in this study (SM00256). Exp. 1 was conducted at the Southwest University of Science and Technology’s research center. Exp. 2 was conducted at the Sichuan Dekon Group Swine Breeding Co., Ltd. (Chengdu, China), a commercial swine production farm with over 12,000 sows.

### Exp. 1

#### Animals and experimental design

The objective of Exp. 1 was to evaluate the effects of dietary supplementation with CoQ10 on the embryonic survival of young (Y) or high-parity (H) sows. A total of 60 postweaning Landrace × Yorkshire (LY) sows, comprising 30 that had completed their 2^nd^ parity and 30 that had completed their 10^th^ parity, were obtained from the Sichuan Dekon Group Swine Breeding Co., Ltd, and kept at the research center of the Southwest University of Science and Technology. Briefly, the Y sows had farrowed an average of 27.8 total born piglets in their past two parities and the H sows had delivered an average of 126.8 total born piglets in their past ten parities. The sows were transferred to individual gestation stalls (2.20 m × 0.65 m) after weaning. Each sow was provided with 3.8 kg of the basic experimental feed (Table 1) per day from post-weaning to mating, and the wean-to-estrus interval of those sows ranged from 4 to 7 d in this study. During this period, the post-weaning sows were checked for the onset of estrous, and were then artificially inseminated twice at 12 h intervals after the occurrence of standing heat (designated as d 0 of gestation) with fresh, pooled semen from three Duroc boars, with each dose containing 3 × 10^9^ spermatozoa. Thin sows were excluded in order to minimize the negative effects of excess tissue mobilization during the previous lactation on their subsequent reproductive performance. Based on a 2 × 2 factorial arrangement of treatments, both Y and H sows were allocated to one of two dietary treatments: a control diet (CON) or a diet supplemented with 1 g/kg CoQ10 (+ CoQ10). The daily intake amount of CoQ10 was based on the relevant literature conducted in mice [12], poultry [13], and stallions [14]. The gestational diet (Table 1) was formulated from corn, soybean meal, wheat bran, and rice bran to provide 3.05 Mcal/kg of digestible energy (DE), 12.55% crude protein (CP), and 0.50% standardized ileal digestible (SID) lysine, respectively [15]. Trace minerals and vitamins were included to meet the requirements of sows as suggested by the National research council (2012) [16]. CoQ10 was obtained from Kingdomway Co., Ltd. (Xiamen, China) with a purity of 99%. Dietary treatments began on the day after mating (d 1) and lasted throughout early gestation until day 28 of gestation. Both Y and H sows were provided with 2.4 kg of feed per day (Table 1), in two equal meals at 08:30 and 14:30. Sows had free access to water, and the temperature was controlled between 18–22 °C. Sows were weighed at d 1 and 28 of gestation.

**Table 1:** Formulation of experimental diets

Ingredients	Content, g/kg	Calculated nutrient	Content, % or indicated units
Corn	597	Digestible energy, Mcal/kg	3.05
Soybean meal (43% CP)	85	Crude protein^c^	12.55
Wheat bran (15% CP)	240	Calcium^c^	0.61
Rice bran	50	Total phosphorus^c^	0.53
*L*-Lysine sulfate (98.5%)	1	STTD phosphorus	0.28
Limestone	8	SID-Lysine	0.50
Calcium phosphate	10	SID-(Met + Cys)	0.37
Sodium chloride, feed-grade	3.5	SID-Threonine	0.35
Choline chloride (50%)	1.5	SID-Tryptophan	0.11
Vitamin premix^a^	0.5		
Trace mineral premix^b^	3.5		
Total	1,000		

*CP* Crude protein, *STTD* Standard total tract digestibility, *SID* Standard ileal digestibility

^a^The vitamin premix provided (per kg of diet): VA, 4,000 IU; VD_3_, 800 IU; VK_3_, 0.5 mg; VE, 44 IU; Thiamine, 1 mg; Riboflavin, 3.75 mg; VB_6_, 1 mg; VB_12_, 15 μg; Niacin, 10 mg; Biotin, 0.2 mg; Pantothenic acid, 12 mg; Folic acid, 1.3 mg

^b^The mineral premix provided (per kg of diet): Fe, 80 mg as FeSO_4_; Cu, 10 mg as CuSO_4_·5H_2_O; I, 0.14 mg as KI; Zn, 100 mg as ZnSO_4_; Se, 0.30 mg as Na_2_SeO_3_; Mn, 25 mg as MnSO_4_

^c^Analyzed value

### Sample collection

Fasting blood samples were collected from each sows’ ear vein and placed in heparin sodium vacutainer tubes on d 1 and 28 of gestation, and plasma was harvested by centrifuging the blood samples for 30 min at 3,000 × *g* and 4 °C. On day 28 of gestation, sows were injected intravenously with compound xylazine (20 μL/kg body weight) and then bled. Slaughter was carried out after the sows had no heartbeat. The uterus and ovaries were removed from each sow immediately after slaughter. Conception rate was calculated as the percentage of successfully pregnant sows. Ovulation was investigated by counting the number of corpora lutea (CL) on both ovaries. The gravid uteri were removed to measure the weight and length of each horn. The number, weight, and crown-rump length of each fetus were recorded, and intra-litter variation was calculated. A fetus was considered to be viable or non-viable according to its appearance within the amnion, a bloody embryo being regarded as a non-viable fetus [17]. The rate of embryonic survival was calculated as the percentage of CL resulting in embryos [18]. Embryonic mortality was defined as the number of CL which failed to produce an embryo. Samples of the endometrium corresponding to the first fetus of the left uterus were separated from the myometrium as previously described [17]. Endometrium samples were snap-frozen in liquid nitrogen and stored at −80 °C for further analysis.

### Measurement of plasma metabolites and hormones

The concentrations of 17β-estradiol (E_2_) and progesterone (P_4_) were determined using a commercially available ELISA kit for E_2_ (R&D Systems, Bio-Techne, Minneapolis, MN, USA) and P_4_ (Abnova Corporation, Walnut, CA, USA). The plasma total CoQ10 level was measured using a competitive ELISA kit, according to the manufacturer’s instructions (abs580226; Absin, Shanghai, China). In brief, a 100-μL plasma sample was placed into 0.9 mL assay buffer, mixed, and then centrifuged for 10 min at 10,000 × *g* and 4 °C. The supernatants were placed into a new centrifuge tube for measurement of the absorbance at 620 nm.

### Measurement of gene expression levels

The relative expressions of genes encoding key enzymes responsible for the de novo synthesis of CoQ10, including decaprenyl diphosphate synthase subunit 1 (*PDSS-*1), and coenzymes (*COQ)-2, COQ-3,* and *COQ*-*6*, as well as regulators in the respiratory chain (peroxisome proliferator-activated receptor gamma coactivator 1-alpha *(PGC1α)*, cytochrome c oxidase (*CcOX*) *I, CcOX IV,* and *CcOX V* were measured using quantitative real-time PCR as previously described [19]. In brief, total RNA was extracted from endometrium tissues using TRIzol reagent (Sigma, Saint Louis, MO, USA), followed by estimation of RNA purity and concentration using a Nanodrop ND-1000 (Nanodrop Technologies, Thermo Fisher Scientific, Wilmington, DE, USA). cDNA was synthesized from 500 ng of total RNA using an iScript™ cDNA synthesis kit (Bio-Rad, Hercules, CA, USA). Quantitative real-time PCR was then performed using a 7900HT Fast Real-Time PCR System (Thermo Fisher Scientific) with SYBR green reagent (RR820A, TaKaRa, Tokyo, Japan). The primers for target genes are shown in Table 2. The threshold cycle (2^–ΔΔCt^) method was used to calculate relative gene expression. β-actin (*ACTB*, Cell Signaling Technology, Boston, USA) was used as a reference gene to normalize the gene expression data.

**Table 2:** Primers for target genes in Exp. 1

Genes	Accession number	Primers (5' → 3')	Tm, °C	Product size, bp
*COQ-2*	XM_003129357.5	F: CACCTACCCGCTAATGAAA	53.5	131
		R: AAGAGGAAGGCAAACAGAT		
*COQ-3*	XM_021090298.1	F: CGATGTGGCGAGGCTGTAAG	61.1	133
		R: CGCGACCTCCAGACACACTC		
*COQ-6*	XM_001929106.5	F: TGATTGGTGCTGATGGTC	52.8	118
		R: TCCGTGGGCTCTGATAAA		
*PDSS-1*	XM_005668142.3	F: CAGATGGGCAAACCGACCT	59.06	80
		R: GGAAACTGCTGACAAGCGAA		
*PGC1α*	NM_213963	F: CCCGAAACAGTAGCAGAGACAAG	60.5	111
		R: CTGGGGTCAGAGGAAGAGATAAAG		
*CcOX I*	AJ950517.1	F: ATTATCCTGACGCATACACAGCA	60.2	127
		R: GCAGATACTTCTCGTTTTGATGC		
*CcOX IV*	AK233334.1	F: CCAAGTGGGACTACGACAAGAAC	60.0	131
		R: CCTGCTCGTTTATTAGCACTGG		
*CcOX V*	AY786556.1	F: ATCTGGAGGTGGTGTTCCTACTG	61.2	160
		R: GTTGGTGATGGAGGGGACTAAA		
*ACTB*	XM_003124280.3	F: TCTGGCACCACACCTTCT		
		R: TGATCTGGGTCATCTTCTCAC		

*COQ-2* Coenzyme Q2, *COQ-3* Coenzyme Q3, *COQ-6* Coenzyme Q6, *PDSS-1* Decaprenyl diphosphate synthase subunit 1, *PGC1α* Peroxisome proliferator-activated receptor gamma coactivator 1-alpha, *CcOX* Cytochrome c oxidase

### Antioxidant capacities of plasma and tissues

Plasma and endometrium samples were used to measure the activities or contents of enzymatic and non-enzymatic antioxidants, including glutathione peroxidase (GSH-Px), malondialdehyde (MDA), catalase (CAT), and superoxide dismutase (SOD), using the relevant commercially available ELISA kits (Nanjing Jiancheng Bioengineering Institute, Nanjing, China), according to the manufacturer’s instructions. The lysates in the endometrium samples were prepared by homogenizing 100 mg tissue samples with 500 μL of lysis buffer (Beyotime Biotechnology, Jiangsu, China) supplemented with a protease inhibitor cocktail (Roche, Indianapolis, IN, USA), followed by centrifuging the homogenized lysates for 20 min at 10,000 × *g* and 4 °C to collect the supernatant. The protein contents of the supernatants of endometrium lysates were detected using a BCA protein assay kit (Thermo Fisher Scientific, Wilmington, DE, USA) on a plate reader, and were then used to normalize the antioxidant parameters.

### Exp. 2

#### Animals and experimental design

The aim of Exp. 2 was to investigate the effects of dietary CoQ10 supplementation of sows with different parities throughout gestation on their reproductive performance. A total of 314 LY crossbred post-weaning sows with 2–9 parities were selected and kept at the gestation facility of the Sichuan Dekon Group Swine Breeding Co., Ltd. During the wean-to-estrus period, sows were fed a common gestating basic diet (Table 1) and checked for the onset of post-weaning estrous. Then sows were artificially inseminated twice with fresh semen from 3 Duroc boars with each dose containing 3 × 10^9^ spermatozoa after they returned to estrous. Sows were then housed individually in gestation stalls (2.25 m × 0.7 m). After mating (designated as day 0 of gestation), sows were randomly allocated to one of two dietary treatments (the basic CON diet, or the supplemented + CoQ10 diet) according to their parities. Both the CON and + CoQ10 diets were the same gestating diet as those used in Exp. 1 (Table 1). All of the sows were provided with 2.4 kg of diets per day in two equal feeds at 08:00 and 16:00 until the end of the experiment (end of farrowing). Access to water was freely available and the temperature was controlled between 20–24 °C.

#### Measurements of reproductive performance

Backfat thickness was measured at the P_2_ position, 60–80 mm from the body midline at the level of the last rib, on d 0 (mating), 30, 60 and 90 of gestation, and at farrowing, using a Digital Backfat Indicator (Renco Corporation, Minneapolis, MN, USA). All of the sows were allowed to farrow naturally without administering hormones. During the farrowing process, the sows were carefully monitored to register the onset of farrowing. Sows with birth intervals exceeded 1 h were given farrowing assistance (FA). The birth interval was calculated as farrowing duration divided by litter size. The litter traits, including the total number of piglets born (TB), the number born alive (BA), and the number of stillborn piglets, were monitored and recorded. The intra-litter uniformity of the newborn piglets (CV) was calculated. The total birthweight was determined as the weight of all of the piglets, once the last one had been delivered. Sows with a litter size less than six were considered unviable as farrowing stock.

#### Sample collection and measurements

Plasma samples (*n* = 11–13 per group) were collected from blood samples taken before the morning meal on d 30 and 100 of gestation. Plasma was obtained by centrifuging the blood samples for 30 min at 3,000 × *g* and 4 °C, and then stored at −20 °C for future analysis. The concentrations of E_2_ and P_4_, and the activities or contents of GSH-Px, MDA, CAT, and SOD were measured using the corresponding commercially available ELISA kits, as previously described for Exp. 1.

### Statistical analysis

Both the normality and homogeneity of variance of the data were evaluated and confirmed using the Shapiro–Wilk and Levene tests, respectively. Data were analyzed as a two-factorial arrangement using the MIXED procedure in SAS 9.4 (SAS Institute Inc., Cary, NC, USA), with parity, diet, and the interaction between them as the fixed effects. The sow nested within group as a random effect. Sows that did not fall pregnant were excluded from the analysis. Individual sow was considered to be the experimental units for all analyses. The differences among treatment means were separated using the least significant difference test. A Chi-square test was used to analyze the percentage of culled sows between groups. Statistical significance was set at *P* < 0.05. A trend was considered at 0.05 < *P* < 0.1.

## Results

### Exp. 1

#### Effect of dietary CoQ10 supplementation on embryonic survival

In Exp. 1, two Y sows (1 in the CON group and 1 in the + CoQ10 group) and seven H sows (3 in the CON group and 4 in the + CoQ10 group) were not pregnant and were excluded from the analysis. The effect of dietary CoQ10 supplementation on embryonic survival is shown in Table 3. Compared with the Y sows, H sows had a greater average BW at mating and at slaughter (*P* < 0.05) but had a lower average daily gain (ADG) between d 1–28 of gestation (*P* < 0.05). The conception rate of H sows tended to be less than that for Y sows (*P* = 0.077) and was not affected by dietary CoQ10 supplementation or the interaction between parity and diet (*P* > 0.05). The H sows had similar numbers of CL as Y sows. The H sows had fewer total embryos and live embryos, and a lower embryonic survival rate (*P* < 0.05) than Y sows. Embryo weight tended to be greater in H sows than in Y sows (*P* = 0.061). Dietary CoQ10 supplementation increased the number of live embryos (*P* = 0.068), embryonic survival, the CV by embryo weight (*P* < 0.05), and the CV by embryo length (*P* < 0.05). Embryonic mortality was significantly higher in H sows than in Y sows (*P* < 0.05). Dietary CoQ10 supplementation reduced the embryonic mortality and number of non-viable embryos (*P* < 0.05). Meanwhile, embryonic survival was affected by the interaction between parity and diet (*P* = 0.087). P_4_ and E_2_ concentrations in sows were not affected by either parity, diet, or their interaction (*P* > 0.05, Table 4).

**Table 3:** Effect of dietary CoQ10 supplementation on embryonic survival in sows in Exp. 1

	Young sows		High-parity sows		SD	*P-*value		
	**CON**	** + CoQ10**	**CON**	** + CoQ10**		**P**	**D**	**P × D**
No. of sows	14	14	12	11	-	-	-	-
BW at mating, kg	185.1^b^	184.7^b^	205.6^a^	204.0^a^	10.8	*	ns	ns
BW at 28 d, kg	198.9^b^	198.4^b^	217.4^a^	216.0^a^	10.2	*	ns	ns
ADG, g/d	492.4^a^	490.9^a^	423.0b	425.5^b^	57.4	*	ns	ns
Conception rate, %	93.3	93.3	80.0	73.3	35.9	0.077	ns	ns
Plasma CoQ10, μmol/L	1.40^b^	2.03^a^	0.94^c^	1.98^a^	0.42	*	*	0.095
Embryo traits								
Copora lutea	21.0	20.4	19.5	20.6	3.80	ns	ns	ns
Total embryo	17.6	17.6	14.5	16.8	3.32	*	ns	ns
Alive embryo	16.9^a^	17.2^a^	13.3^b^	16.4^ab^	3.00	*	0.068	ns
Embryonic mortality	4.1^b^	3.2^b^	6.2^a^	4.3^ab^	1.92	*	*	ns
Non-viable embryos	0.64	0.43	1.17	0.45	0.76	ns	*	ns
Embryonic survival, %	81.0^a^	84.7^a^	68.8^b^	79.3^a^	6.93	*	*	0.087
Embryo weight	2.0	2.1	2.2	2.1	0.22	0.061	ns	ns
Embryo weight CV, %	12.6^a^	10.8^ab^	12.6^a^	10.0^b^	2.06	ns	*	ns
Embryo length	26.4	25.8	26.8	26.1	2.57	ns	ns	ns
Embryo length CV, %	6.2	5.2	6.3	5.2	1.52	ns	*	ns

*BW* Bodyweight, *ADG* Average daily weight gain, *CV* Intra-litter variation, *P* Parity, *D* Diet, *P* × *D* Interaction between parity and diet

^a–c^Means in the same row with different superscripts differ significantly (*P* < 0.05)

^*^P < 0.05; ns, non-significant, *P* > 0.10. Trend was considered at 0.05 < *P* < 0.1

**Table 4:** Effect of dietary CoQ10 supplementation on hormone secretion of E_2_ and P_4_ in young and high-parity sows in Exp. 1

	Young sows		High-parity sows		SD	*P*-value		
	**CON**	** + CoQ10**	**CON**	** + CoQ10**		**P**	**D**	**P × D**
No. of sows	14	14	12	11	-	-	-	-
E_2_, pg/mL	308.2	298.4	289.4	311.6	163.2	ns	ns	ns
P_4_, ng/mL	22.9	28.4	27.4	28.6	16.7	ns	ns	ns

*CON* Control diet, + *CoQ10* Control diet supplemented with 1 g/kg coenzyme Q10, *E*_*2*_ 17β-estradiol, *P*_*4*_ Progesterone, *P* Parity, *D* Diet, *P* × *D* Interaction between parity and diet, *ns* non-significant, *P* > 0.10

#### Effect of dietary CoQ10 supplementation on antioxidant properties

The effect of dietary CoQ10 supplementation on the antioxidant properties of sows on d 28 of gestation are shown in Table 5. The H sows had greater concentrations of MDA in the plasma and endometrium samples, lower concentrations of SOD in the plasma, and lower GSH-Px activity in the endometrium (*P* < 0.05). Dietary CoQ10 supplementation decreased the concentrations of MDA in the plasma and endometrium samples (*P* < 0.05) and increased the activity of SOD in the plasma (*P* < 0.05) and the activity of GSH-Px in the plasma and the endometrium (*P* < 0.05). Antioxidant properties were not affected by the interaction between parity and diet (*P* > 0.05).

**Table 5:** Effect of dietary CoQ10 supplementation on antioxidant properties in plasma and endometrium samples in Exp. 1

	Young sows		High-parity sows		SD	*P-*value		
	**CON**	** + CoQ10**	**CON**	** + CoQ10**		**P**	**D**	**P × D**
No. of sows	14	14	12	11	-	-	-	-
In plasma								
GSH-Px, U/mL	993.3^ab^	1,095.7^a^	726.7^b^	1,023.6^ab^	227.6	*	*	ns
CAT, U/mL	14.5	16.3	13.1	14.9	4.89	ns	ns	ns
MDA, nmol/mL	1.94^b^	1.66^b^	2.53^a^	1.73^b^	0.56	*	*	ns
SOD, U/mL	20.8^ab^	25.2^a^	16.2^b^	22.0^ab^	6.21	*	*	ns
In endometrium								
GSH-Px, U/mg	103.2^ab^	115.9^a^	85.3^b^	101.7^ab^	22.3	*	*	ns
CAT, U/mg	9.23^ab^	11.45^a^	8.18^b^	10.84^ab^	2.85	ns	*	ns
MDA, nmol/mg	0.90^ab^	0.70^b^	1.26^a^	0.85^b^	0.36	*	*	ns
SOD, U/mg	13.5	16.4	11.8	13.2	5.70	ns	ns	ns

*CON* Control diet, + *CoQ10* Control diet supplemented with 1 g/kg coenzyme Q10, *GSH-Px* Glutathione peroxidase, *CAT* Catalase, *MDA* Malondialdehyde, *SOD* Superoxide dismutase, *P* Parity, *D* Diet, *P* × *D* Interaction between parity and diet

^a,b^Means in the same row with different superscripts differ significantly (*P* < 0.05)

^*^P < 0.05; *ns*, non-significant, *P* > 0.10

#### Effect of dietary CoQ10 supplementation on gene expression in endometrium samples

**Fig. 1:**
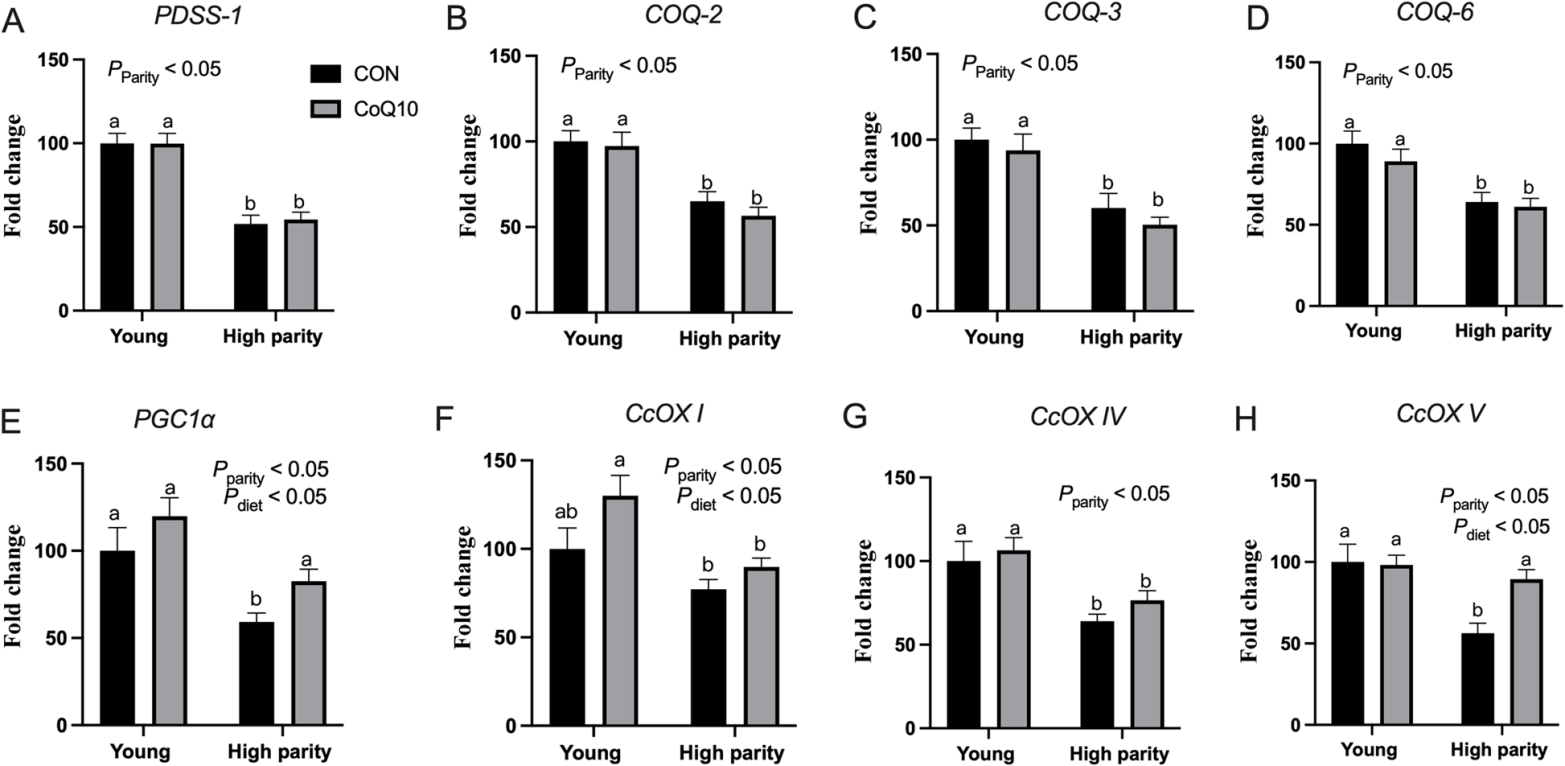
Effects of dietary CoQ10 supplementation on gene expression in endometrium samples in Exp. 1 (*n* = 10). **A**–**D** expressions of *PDSS-1*, *COQ-2*, *COQ-3*, and *COQ-6* mRNA for de novo CoQ10 synthesis. **E**–**H** expressions of *PGC1α*, *CcOX I*, *CcOX IV*, and *CcOX V* mRNA in the regulation of the respiratory chain. *PDSS-1*, decaprenyl diphosphate synthase subunit 1; *COQ-2*, coenzyme Q2; *COQ-3*, coenzyme Q3; *COQ-6*, coenzyme Q6; *PGC1α*, Peroxisome proliferator-activated receptor gamma coactivator 1-alpha; *CcOX*, cytochrome c oxidase

The relative expressions of genes in the endometrium samples are shown in Fig. 1. The relative expressions of the genes *PDSS-1*, *COQ-2*, *COQ-3*, and *COQ-6* were significantly lower in H sows than in Y sows (*P* < 0.05, Fig. 1A–D), but were not affected by dietary CoQ10 supplementation or the interaction between parity and diet (*P* > 0.05). As shown in Fig. 1E–H, the relative expressions of *PGC1α*, *CcOX I*, *CcOX IV*, and *CcOX V* were lower in H sows than in Y sows (*P* < 0.05). In addition, the relative expressions of *PGC1α*, *CcOX I*, and *CcOX V* were elevated by dietary CoQ10 supplementation (*P* < 0.05). No interactions between parity and diet were observed on the expressions of *PGC1α*, *CcOX I*, *CcOX IV*, and *CcOX V.*

### Exp. 2

#### Culling data of sows

As shown in Table 6, a total of 314 LY sows were initially selected, but only 260 of them fell pregnant after one round of artificial insemination. Fourteen of the pregnant sows were culled due to illness, abortion, and lameness, resulting in a final total of 246 farrowed sows. However, the performance data of only 225 sows were used for the analysis because 21 sows had total litter sizes less than six, and were excluded from the study. A Chi-square test was used to analyze the percentage of culled sows between the diet groups, but revealed no statistical difference between them (*P* > 0.05, Table 6).

**Table 6:** Culling data of sows in Exp. 2

	CON								+ CoQ10							
	**2**	**3**	**4**	**5**	**6**	**7**	**8**	**9**	**2**	**3**	**4**	**5**	**6**	**7**	**8**	**9**
Initial	38	34	31	18	10	9	9	8	37	34	32	19	9	10	8	8
Concepted	31	30	27	14	8	8	7	4	32	31	28	14	7	7	6	6
Total farrowed	29	29	26	13	7	7	7	4	31	29	27	13	7	6	6	5
Farrowed effective	27	28	25	11	6	6	5	4	29	26	25	12	5	7	4	5
Total culls	11	6	6	7	4	3	4	4	8	8	7	7	4	3	4	3
Percentage culls, %	28.9	17.6	19.4	38.9	40.0	33.3	44.4	50.0	21.6	23.5	21.9	36.8	44.4	30.0	50.0	37.5

*CON* Control diet, + *CoQ10* Control diet supplemented with 1 g/kg coenzyme Q10

#### Effect of dietary CoQ10 supplementation on sow productive performance

As shown in Table 7, the backfat thickness of sows on d 1, 30, 60, 90, and at farrowing, were not affected either by parity or diet, or by the interaction between them (*P* > 0.05). The effect of dietary CoQ10 supplementation on the reproductive traits of sows are shown in Table 8. The numbers of total piglets born and piglets born alive were affected by the parity of the sows (*P* < 0.05), and peaked at parity 4. The number of stillborn, intrauterine growth restricted (IUGR), average birthweight, intra-litter CV, parturition length, FA percentage, and birth interval were not affected by parity (*P* > 0.05). Dietary CoQ10 supplementation improved the total number of piglets born (*P* = 0.070), born alive, and born effective (*P* < 0.05). Meanwhile, dietary CoQ10 supplementation decreased the intra-litter CV (*P* < 0.05) and the percentage of sows requiring FA (*P* = 0.041). No interactive effect between parity and diet was observed on these parameters. In addition, secretions of the hormones E_2_ and P_4_ were not affected by either parity or diet, or the interaction between them (*P* > 0.05, Table 9).

**Table 7:** Backfat thickness of sows during gestation in Exp. 2, mm

	CON					+ CoQ10					SD	*P-*value		
	**2**	**3**	**4**	**5**	**6–9**	**2**	**3**	**4**	**5**	**6–9**		**P**	**D**	**P × D**
No. of sows	27	28	25	11	21	29	26	25	12	21	-	-	-	-
1 d	14.4	14.6	13.3	14.0	13.5	14.5	13.7	14.6	13.7	14.3	3.10	ns	ns	ns
30 d	15.4	15.1	14.2	14.0	14.2	15.1	14.3	15.0	14.0	15.3	2.95	ns	ns	ns
60 d	16.4	16.4	15.4	15.1	15.7	16.3	15.5	16.0	15.3	16.0	2.85	ns	ns	ns
90 d	16.8	17.3	16.9	15.7	16.7	17.5	16.5	16.6	16.0	16.8	2.80	ns	ns	ns
At farrowing	17.3	17.9	17.5	17.5	17.5	18.2	17.1	17.0	18.3	17.6	2.83	ns	ns	ns

*CON* Control diet, + *CoQ10* Control diet supplemented with 1 g/kg coenzyme Q10, *P* Parity, *D* Diet, *P* × *D* Interaction between parity and diet, *ns* non-significant, *P* > 0.10

**Table 8:** Effect of dietary CoQ10 supplementation on the reproductive performance of sows in Exp. 2

	CON					+ CoQ10					SD	*P-*value		
	**2**	**3**	**4**	**5**	**6–9**	**2**	**3**	**4**	**5**	**6–9**		**P**	**D**	**P × D**
No. of sows	27	28	25	11	21	29	26	25	12	21	-	-	-	-
Total born	13.74^ab^	14.29^ab^	14.36^ab^	13.45^ab^	12.05^b^	13.72^ab^	14.96^ab^	15.20^a^	14.25^ab^	13.67^ab^	3.08	*	0.070	ns
Born alive	12.52^ab^	12.6^ab^	13.20^ab^	12.45^ab^	10.71^b^	12.45^ab^	14.04^a^	13.76^ab^	13.17^ab^	12.90^ab^	2.93	*	*	ns
Stillborn	1.22	1.61	1.16	1.00	1.33	1.28	0.92	1.44	1.08	0.86	1.57	ns	ns	ns
NO. of IUGR	0.63	0.89	0.72	0.64	0.62	0.34	0.46	0.80	1.00	0.38	0.78	ns	ns	ns
NO. effective	11.89	11.79	12.48	11.82	10.10	12.10	13.58	12.96	12.17	12.52	2.83	ns	*	ns
Birthweight, kg	1.45	1.40	1.45	1.53	1.46	1.51	1.41	1.41	1.40	1.44	0.23	ns	ns	ns
Intra-litter CV, %	18.0	20.2	20.5	19.2	19.8	17.6	16.4	18.7	19.6	17.3	0.06	ns	*	ns
Parturition length, min	237.5	299.3	310.8	253.7	272.0	264.6	290.3	289.3	277.5	230.7	144.3	ns	ns	ns
Birth interval, min	17.5	23.4	21.9	20.2	23.4	19.6	21.0	19.6	19.9	17.4	10.0	ns	ns	ns
FA, %	40.7	53.6	44.0	54.5	66.7	31.0	34.6	36.0	50.0	42.9	49.7	ns	0.041	ns

*CON* Control diet,  + *CoQ10* Control diet supplemented with 1 g/kg coenzyme Q10, *P* Parity, *D* Diet, *P* × *D* Interaction between parity and diet. *IUGR*, intrauterine growth restriction, *CV* Intra-litter variation, *FA* Farrowing assistance

^a,b^Means in the same row with different superscripts differ significantly (*P* < 0.05)

^*^P < 0.05; ns, non-significant, *P* > 0.10. Trend was considered at 0.05 < *P* < 0.10

**Table 9:** Effect of dietary CoQ10 supplementation on the hormone secretions of sows in Exp. 2

	CON					+ CoQ10					SD	*P-*value		
	**2**	**3**	**4**	**5**	**6–9**	**2**	**3**	**4**	**5**	**6–9**		**P**	**D**	**P × D**
No. of sows	12	12	12	11	13	12	12	12	12	13	-	-	-	-
E_2_, pg/mL	289.3	297.8	332.1	309.3	337.5	311.4	313.9	298.4	299.6	302.1	141.2	ns	ns	ns
P_4_, ng/mL	32.9	29.8	33.7	31.8	30.5	28.4	28.5	32.8	32.2	31.3	12.6	ns	ns	ns

*CON* Control diet,  + *CoQ10* Control diet supplemented with 1 g/kg coenzyme Q10, *E*_*2*_ 17β-estradiol, *P*_*4*_ Progesterone, *P* Parity, *D* Diet, *P* × *D* Interaction between parity and diet. *ns* non-significant, *P* > 0.10

#### Effect of dietary CoQ10 supplementation on the antioxidant properties of sows

As shown in Table 10, the plasma CoQ10 levels of sows on day 30 (*P* < 0.05) and day 100 (*P* = 0.073) of gestation decreased with rising parity, and were increased by dietary CoQ10 supplementation (*P* < 0.05). With increasing parity, sows had increased plasma levels of MDA on day 30 d and day 100 of gestation (*P* < 0.05). Dietary CoQ10 supplementation significantly increased the activities of GSH-Px and SOD in the plasma and decreased the plasma MDA content (*P* < 0.05). The interaction between parity and diet exerted no effect on the levels of antioxidant properties in the plasma (*P* > 0.05).

**Table 10:** Effect of dietary CoQ10 supplementation on plasma concentrations of antioxidant parameters of sows in Exp. 2

	CON					+ CoQ10					SD	*P-*value		
	**2**	**3**	**4**	**5**	**6–9**	**2**	**3**	**4**	**5**	**6–9**		**P**	**D**	**P × D**
No. of sows	12	12	12	11	13	12	12	12	12	13	-	-	-	-
Plasma CoQ10, μmol/L														
30 d	1.52^abc^	1.40^abc^	1.26^abc^	1.06^bc^	0.85^c^	1.97^a^	1.74^ab^	1.73^ab^	1.82^a^	1.64^ab^	0.58	*	*	ns
100 d	1.52^abcd^	1.46^bcd^	1.47^bcd^	1.33^ cd^	1.20^d^	2.25^ab^	2.05^abc^	2.25^a^	2.20^ab^	1.70^abcd^	0.60	0.073	*	ns
Plasma GSH-Px, U/mL														
30 d	872.6^ab^	858.8^ab^	752.6^b^	768.3^ab^	716.1^b^	1144.3^a^	971.2^ab^	868.4^ab^	996.1^ab^	928.3^ab^	280.7	ns	*	ns
100 d	878.1^ab^	789.1^ab^	800.5^ab^	711.3^b^	741.6^b^	1,005.1^ab^	1,102.1^a^	981.5^ab^	980.6^ab^	947.1^ab^	267.9	ns	*	ns
CAT, U/mL														
30 d	34.32	34.73	32.49	34.72	29.82	34.83	27.85	33.95	32.50	29.87	11.8	ns	ns	ns
100 d	36.58	31.99	34.83	34.77	33.46	33.68	32.23	37.99	33.47	35.12	13.8	ns	ns	ns
MDA, nmol/mL														
30 d	2.80^abcd^	3.10^abcd^	3.49^abc^	3.75^ab^	4.14^a^	1.82^d^	2.14^ cd^	2.40^bcd^	2.52^bcd^	2.66^bcd^	1.03	*	*	ns
100 d	2.51^bcd^	3.07^abc^	3.61^ab^	4.01^a^	4.07^a^	1.94^ cd^	1.54^d^	2.74^abcd^	2.76^abcd^	2.98^abc^	1.08	*	*	ns
SOD, U/mL														
30 d	48.80^ab^	44.08^ab^	44.26^ab^	49.05^ab^	41.51^b^	51.07^ab^	50.88^ab^	49.87^ab^	59.46^a^	48.15^ab^	12.9	ns	*	ns
100 d	59.39	50.64	57.08	60.78	50.31	63.44	61.92	63.22	67.55	61.38	14.8	ns	*	ns

*CON* Control diet, + *CoQ10* Control diet supplemented with 1 g/kg coenzyme Q10, *GSH-Px* Glutathione peroxidase, *CAT* Catalase, *MDA* Malondialdehyde, *SOD* Superoxide dismutase, *P* Parity, *D* Diet, *P* × *D* Interaction between parity and diet, *IUGR* Intrauterine growth restriction

^a–d^Means in the same row with different superscripts differ significantly (*P* < 0.05)

^*^P < 0.05; ns, non-significant, *P* > 0.10. Trend was considered at 0.05 < *P* < 0.1

## Discussion

The annual culling rate of sows reaches about 50% in swine production systems, and is mainly due to culling in response to defects in reproductive performance [2]. Currently, the proportion of sows with high parity (> 6) in swine production systems is usually below 10% and these animals are usually referred to as high-parity sows. However, these H sows still have reproductive potential because they can ovulate similar (or even greater) numbers of mature oocytes compared with Y (parity 2 or 3) sows [20]. The H sows are more likely to be culled due to their small litter sizes [2], possibly due to uterine dysfunction inducing the loss of early embryos, because development of the uterus tends to become more variable when sows advance to higher parities [21]. In this study, we compared the ovulation rate of Y and H sows by counting the number of CL on their ovaries and found similar numbers of ovulations in both groups, a result in agreement with observations made on a swine farm in the Netherlands [20].

However, the total numbers of live embryos were lower in H sows than in Y sows. Furthermore, the number of embryonic mortalities, as shown by the number of CL that failed to develop into an embryo, were significantly higher in H sows. The lower number of living embryos in H sows resulted in a lower embryonic survival rate compared with Y sows, suggesting that the decreased litter size in H sows could be largely due to greater losses of early embryos. This embryonic loss was not attributed to hormone induced physiological dysfunction because the concentrations of circulating P_4_ and E_2_, two key hormones mediating embryonic implantation in pigs [22], were not affected by parity.

In order to better understand the reason(s) why embryonic survival declined with parity, we focused on the bioenergetic state of the reproductive tissues because reproduction is an energy-dependent process which requires a continuous supply of ATP for cellular metabolism [3]. Studies on humans have revealed that the age-dependent decline in fecundity [23] was associated with declining cellular ATP provision to reproductive organs such as the ovaries [24]. However, details of the age-dependent decline in cellular function in the reproductive system of sows in actual swine production units remain largely unclear.

CoQ10 is one of most important organic antioxidant compounds present in most cellular membranes and is required to transfer electrons from complexes I and II to complex III in the respiratory chain in the mitochondrial inner membrane [25–27]. Interestingly, recent studies have revealed that the *PDSS*-2 and *CoQ-6* genes encoding the key enzymes responsible for the de novo synthesis of CoQ10, decreased in the ovaries of older females in both mice and humans [6, 7]. However, it remains unclear whether there is an age-dependent decline of CoQ10 synthesis in porcine reproductive tissues. We measured the plasma concentrations of total CoQ10 in sows with different parities and found that H sows have lower concentrations of CoQ10 than Y sows. Because the ovulation rate did not differ between Y and H sows in this study, we hypothesize that the quality of uterine development, rather than ovarian follicular development, could be involved in the control of embryonic survival. Endometrium tissues were collected from the uterus to determine the levels of the key enzymes responsible for the de novo synthesis of CoQ10 and found that mRNA expressions of *PDSS-1, COQ-2, COQ-3,* and *COQ-6* were significantly downregulated in H sows compared with Y sows. Disruption of CoQ10 synthesis has been observed to be associated with the malfunction of mitochondria in reproductive tissues, such as the ovaries and uterus [6, 7, 28]. As a marker for oxidative stress, the MDA contents of the plasma and endometrium samples were significantly elevated in H sows compared with Y sows. On the contrary, the enzymatic activities of GSH-Px, whose main biological function is to reduce lipid hydroperoxides and free hydrogen peroxide to protect the cellular functions from oxidative damage, were significantly lower in H sows than in Y sows. These results show that the embryonic deaths in H sows were associated with impairment of de novo CoQ10 synthesis and the redox balance.

CoQ10 is a powerful natural antioxidant that maintains the cellular redox balance to prevent cell senescence due to oxidative stress. Therefore, we tested whether the age-dependent decline in embryonic survival of gestating sows could be prevented by dietary CoQ10 supplementation. In this study, dietary CoQ10 was added at a level of 1 g/kg diet (~ 12.5 mg/kg BW per day), a dose sufficient to cause an increase in circulating CoQ10 concentration with beneficial physiological changes, as shown in humans [29, 30], stallions [14], guinea pigs [31], mice [12], and laying hens [13]. In this study, dietary CoQ10 supplementation increased the circulating CoQ10 level in both experiments, indicative of effective dietary treatment. Dietary CoQ10 supplementation did not affect the conception rate or the ovulation rate, possibly because both are mainly affected by the competence of oocyte development [32], and the dietary treatment started from mating rather than from the follicular developmental phase. One of the most important findings of this study was that sows receiving dietary CoQ10 supplementation had higher live embryo and embryonic survival, while the incidence of embryonic mortality and non-viable embryos decreased significantly. In particular, the negative effects of aging on the total numbers of embryos, numbers of live embryos, and embryonic survival of H sows were reversed by dietary CoQ10 supplementation, suggesting that dietary CoQ10 supplementation could improve the embryonic survival of sows. Dietary CoQ10 supplementation was associated with improved cellular energy metabolism and redox balance. Dietary CoQ10 supplementation did not affect the relative expressions of genes relating to de novo CoQ10 synthesis, suggesting that dietary CoQ10 supplementation did not affect CoQ10 production. The *PGC1α*, *CcOX I*, *CcOX IV*, and *CcOX V* genes regulate the functional cellular energy metabolism of various tissues, including the reproductive tracts [33]. However, the relative expressions of *PGC1α*, *CcOX I*, *CcOX IV*, and *CcOX V* were lower in H sows than in Y sows. Interestingly, in this study the decreased expressions of *PGC1α*, *CcOX I*, *CcOX IV*, and *CcOX V* in H sows were reversed by a similar degree to those in Y sows, suggesting that dietary CoQ10 supplementation could enhance cellular energy metabolism in the uterus tissue. Dysfunction of the respiratory chain is usually associated with imbalanced oxidative stress. In line with this, the decrease of GSH-Px activity and increase of MDA content of H sows were reversed to similar levels as those in Y sows. These results showed that dietary CoQ10 could reverse the negative effects of age-dependent increases in oxidative stress and embryonic survival.

In actual swine production, litter sizes begin to decline after parity 4 [34]. The findings of Exp. 1 revealed that dietary CoQ10 supplementation could improve embryonic survival in gestating sows with high parity. Therefore, we recommend that dietary CoQ10 supplementation should be trialed in an actual sow production system to test its effect on the reproductive performance of gestating sows with different parities in a real-world situation. In this study, the culling percentage of sows was not affected by dietary CoQ10 supplementation. In addition, dietary CoQ10 supplementation did not affect the backfat thickness of sows with different parities. Interestingly, the reproductive traits, such as the total number of piglets born, and the number of piglets born alive and born effective, significantly increased with age before parity 4, and then decreased with increasing parity, indicating an age-dependent decline of reproductive performance [34]. In particular, dietary CoQ10 supplementation of sows with parity 6–9 increased the total number of piglets born, and the number born alive and born effective per sow by 1.62, 2.19, and 2.42 compared with those fed the CON diet, respectively. Meanwhile, intra-litter CV, a parameter showing the nutrient transfer capacity and functional variation in uterine development [35], was decreased by dietary CoQ10 supplementation. Increased in intra-litter variation can result in preweaning piglet death, variable piglet weights at weaning, and impaired piglet growth after weaning [36]. Taken together, both experiments showed that lower intra-litter variation in embryo weight, can be achieved by dietary CoQ10 supplementation through enhanced uterine and placental development in gestating sows. Interestingly, the improvement of litter traits was associated with improved antioxidant capacity and lower MDA levels, a marker of oxidative stress. These results agree with those of other recent studies, which found that antioxidant capacity could affect the litter size of sows [37–39]. Dietary supplementation of CoQ10 has a positive effect on litter size (or embryo survival rate), but the specific mechanism is still unclear. This requires further research on mining mechanisms.

## Conclusion

The findings of this study showed that dietary CoQ10 can improve the embryonic survival and litter size of gestating sows with high parity, probably by improving the antioxidant capacity of uterine developmental function. This is the first study to show the positive effects of dietary CoQ10 supplementation on sow performance, and provides insights into the improvement of porcine reproductive performance via nutritional means.

## Data Availability

All data generated or analyzed during this study are available from the corresponding author on reasonable request.

## Abbreviations

BA: Born alive
CAT: Catalase
CcOX: Cytochrome c oxidase
CL: Corpora lutea
CON: Control diet
COQ-2: Coenzyme Q2
COQ-3: Coenzyme Q3
COQ-6: Coenzyme Q6
CoQ10: Coenzyme Q10
E_2_: 17β-Estradiol
FA: Farrowing assistance
GSH-Px: Glutathione peroxidase
H sows: High-parity sows
LY: Landrace × Yorkshire
MDA: Malondialdehyde
P_4_: Progesterone
PDSS-1: Decaprenyl diphosphate synthase subunit 1
PGC1α: Peroxisome proliferator-activated receptor gamma coactivator 1-alpha
SOD: Superoxide dismutase
TB: Total born
Y sows: Young sows
